# Supplementary material for: Splice-Junction-Based Mapping of Alternative Isoforms in the Human Proteome
Source: Cell Rep. Author manuscript; Available in PMC 2020 Jan 15. (PMC6961840; doi:10.1016/j.celrep.2019.11.026)
Supplement: 3 [file NIHMS1546469-supplement-3.zip › DF2/PXD0010154/Ovary-25-O00429-DKVASGGGGVGDGVQEPTTGNWR.pdf]

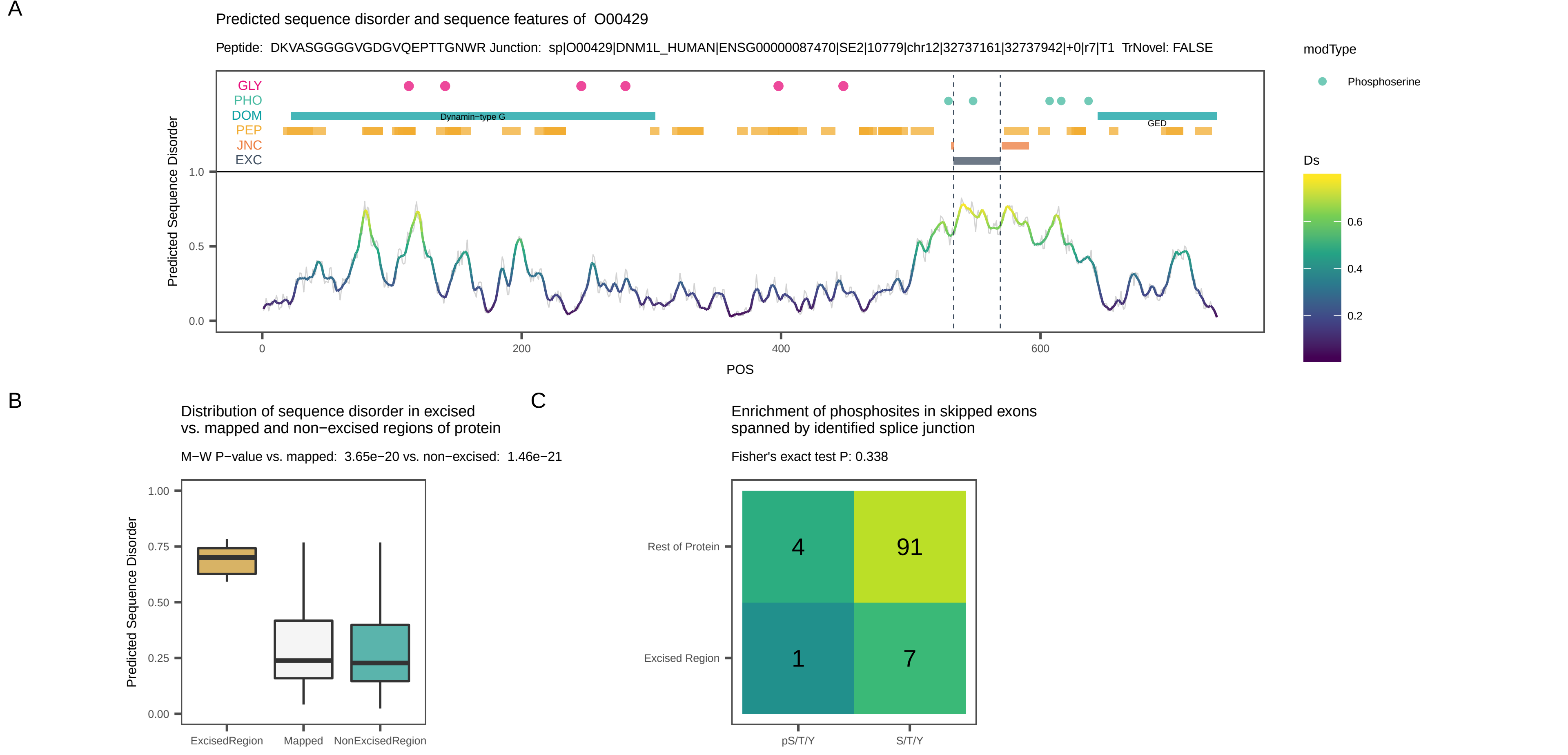

A
Predicted sequence disorder and sequence features of O00429
Peptide: DKVASGGGGVGDGVQEPTTGNWR Junction: sp|O00429|DNM1L_HUMAN|ENSG00000087470|SE2|10779|chr12|32737161|32737942|+0|r7|T1 TrNovel: FALSE
modType
Phosphoserine
GLY
PHO
DOM
PEP
JNC
EXC
Dynamin−type G
GED
Predicted Sequence Disorder
1.0
0.5
0.0
0
200
400
600
POS
Ds
0.6
0.4
0.2
B
Distribution of sequence disorder in excised
vs. mapped and non−excised regions of protein
M−W P−value vs. mapped: 3.65e−20 vs. non−excised: 1.46e−21
Predicted Sequence Disorder
1.00
0.75
0.50
0.25
0.00
ExcisedRegion
Mapped
NonExcisedRegion
C
Enrichment of phosphosites in skipped exons
spanned by identified splice junction
Fisher's exact test P: 0.338
Rest of Protein
Excised Region
4
91
1
7
pS/T/Y
S/T/Y
